# Intrafractional accuracy and efficiency of a surface imaging system for deep inspiration breath hold during ablative gastrointestinal cancer treatment

**DOI:** 10.1002/acm2.13740

**Published:** 2022-07-30

**Authors:** Chuan Zeng, Wei Lu, Marsha Reyngold, John J. Cuaron, Xiang Li, Laura Cerviño, Tianfang Li

**Affiliations:** ^1^ Department of Medical Physics Memorial Sloan Kettering Cancer Center New York New York USA; ^2^ Department of Radiation Oncology Memorial Sloan Kettering Cancer Center New York New York USA

**Keywords:** deep inspiration breath hold, intrafraction motion, pancreatic cancer, surface imaging guidance

## Abstract

**Purpose:**

Beam gating with deep inspiration breath hold (DIBH) usually depends on some external surrogate to infer internal target movement, and the exact internal movement is unknown. In this study, we tracked internal targets and characterized residual motion during DIBH treatment, guided by a surface imaging system, for gastrointestinal cancer. We also report statistics on treatment time.

**Methods and materials:**

We included 14 gastrointestinal cancer patients treated with surface imaging‐guided DIBH volumetrically modulated arc therapy, each with at least one radiopaque marker implanted near or within the target. They were treated in 25, 15, or 10 fractions. Thirteen patients received treatment for pancreatic cancer, and one underwent separate treatments for two liver metastases. The surface imaging system monitored a three‐dimensional surface with ± 3 mm translation and ± 3° rotation threshold. During delivery, a kilovolt image was automatically taken every 20° or 40° gantry rotation, and the internal marker was identified from the image. The displacement and residual motion of the markers were calculated. To analyze the treatment efficiency, the treatment time of each fraction was obtained from the imaging and treatment timestamps in the record and verify system.

**Results:**

Although the external surface was monitored and limited to ± 3 mm and ± 3°, significant residual internal target movement was observed in some patients. The range of residual motion was 3–21 mm. The average displacement for this cohort was 0–3 mm. In 19% of the analyzed images, the magnitude of the instantaneous displacement was > 5 mm. The mean treatment time was 17 min with a standard deviation of 4 min.

**Conclusions:**

Precaution is needed when applying surface image guidance for gastrointestinal cancer treatment. Using it as a solo DIBH technique is discouraged when the correlation between internal anatomy and patient surface is limited. Real‐time radiographic verification is critical for safe treatments.

## 1 INTRODUCTION

Deep inspiration breath hold (DIBH) has been extensively used in motion management for radiation therapy.^1^, ^2^ Moderate or active breathing control DIBH is delivered using an equipment similar to a spirometer,^1^, ^3^ whereas voluntary DIBH usually depends on external surrogates, the most common one being the Varian Real‐time Positioning Management (RPM) system (Varian Medical System, Palo Alto, CA).^4^, ^5^ Last few years, surface image guidance has become an important tool for DIBH.^6^ However, it is still limited by the correlation between internal anatomy and patient surface. This correlation may not be strong enough to indicate its use as a solo technique for treatments using high doses per fraction and small planning target volume (PTV) margins, such as in stereotactic radiosurgery or stereotactic body radiation therapy (SBRT). For workflow considerations, DIBH also needs patient cooperation and additional time, depending on the technique used.^2^ Depending on patient compliance, treatment time has increased relative to standard treatment. Irregular breathing will further slow down the process. Overly long treatment time is inconvenient and may cause setup errors due to movements of an uncomfortable patient.

For locally advanced unresectable pancreatic cancer, SBRT or hypofractionated ablative radiotherapy was associated with durable control of the primary tumor and low toxicity rates, leading to favorable survival outcomes.^7^, ^8^, ^9^, ^10^, ^11^ Ablative radiotherapy combines stereotactic technology with innovative solutions for motion management to reach the target ablation dose while preserving the adjacent luminal gastrointestinal (GI) tract.^11^ Ablation technique gives deliberately inhomogeneous doses with intentional hot spots within the target to achieve sharp dose gradients and spare normal tissues.^10^, ^11^ These sharp dose gradients bring normal tissues near harmful radiation doses. Thus, appropriate organ motion management with emerging techniques is essential to avoid serious complications, enabling curative radiation doses to be given in patients with inoperable disease.^11^, ^12^, ^13^

Published studies on surface‐imaging guided radiotherapy (SGRT) are mainly for breast radiotherapy,^5^, ^14^, ^15^, ^16^ where it has been used extensively in the development of DIBH technique and the external surface are well correlated with the treatment volume.^14^, ^16^ The conclusions drawn from those studies may not be applicable to GI treatments since a target in the abdomen cannot be tracked in the same way. Schaerer et al. used surface imaging with a deformable registration to achieve multidimensional tracking of the abdominal surface.^17^, ^18^ Lauria et al. quantified the use of anterior torso skin surface position measurement as a breathing surrogate using fast helical computed tomography (CT).^19^

At our institution, we have established procedures to apply surface image guidance for intrafractional monitoring to breast and brain treatments.^20^, ^21^ In the present study, we used kilovolt X‐ray imaging during treatment to record the internal target motion in GI cancer patients during DIBH ablation therapy guided by a surface imaging system. Internal targets were tracked through radiopaque markers near or within the lesions using X‐ray images triggered during treatment.^22^, ^23^, ^24^, ^25^ We characterized residual motion as the maximum magnitude of motion observed during each treatment session and analyzed the displacement vectors from the initial setup position to the positions observed in the X‐ray images. We also reported treatment time statistics for patients’ comfort and workflow/workload considerations.

## 2 METHODS

### 2.1 Patients

A data exemption from institutional review board was approved prior to the study. The study included 14 consecutive patients with GI cancer who underwent DIBH radiation treatment with surface‐imaging guidance and on‐treatment triggered kV image monitoring between 2019 and 2021. Every patient had at least one radiopaque marker (fiducial marker, surgical clip from attempted resection, or bile duct stent) near or within the target. The median distance between the markers and the centers of GTVs was 2 cm (range 1–4 cm), compared to median GTV of 40 cc (range 10–300 cc). Table 1 displays the demographic and treatment characteristics of the patients. The median age was 73 years, and the range was 53–87 years. Seven patients received 25 fractions of total dose 75 Gy to gross tumor volume (GTV) and 45 Gy to clinical target volume (CTV); six patients received 15 fractions total dose of 67.5 Gy and 37.5 Gy; and one was treated in 10 fractions to 40 Gy and 30 Gy^9^ (these doses are evaluated prospectively in a clinical trial [NCT03523312]). Volumetrically modulated arc therapy (VMAT) was planned and delivered on TrueBeam linacs (Varian Medical Systems). Those accelerators can use the kilovolt imager installed on the gantry (On‐Board Imager, Varian Medical Systems) to obtain kilovolt X‐ray images during megavolt (MV) beam delivery.^23^

**TABLE 1:** Patient demographic and treatment characteristics

Patient number Age/year Gender			Site	Number of fractions	*d*_marker‐to‐GTV_/cm	GTV/cc
1	75	Male	Pancreas	15	1.10	33.5
2	67	Female	Pancreas	25	1.45	19.6
3	79	Female	Pancreas	25	1.10	42.5
4	56	Female	Pancreas	10	2.12	82.2
5	73	Female	Pancreas	25	3.77	24.3
6	73	Female	Pancreas	15	1.48	7.2
7	63	Male	Ampulla	25	1.54	283.0
8	62	Female	Pancreas	25	1.07	37.8
9	87	Female	Pancreas	15	2.89	178.8
10	73	Male	Pancreas	15	2.35	119.9
11	61	Male	Pancreas	15	2.11	21.5
12a^a^	53	Female	Liver	15	0.36	16.3
12b^a^	53	Female	Liver	15	0.65	42.2
13	79	Female	Pancreas	25	2.01	95.8
14	77	Male	Pancreas	15	2.13	27.2

Abbreviation: GTV, gross tumor volume.

^a^ Patient 12 was treated separately for two liver metastases.

### 2.2 Computed tomography simulation

CT simulations were performed using the Brilliance Big Bore CT simulators (Koninklijke Philips N.V., Amsterdam, Netherlands). Patients were immobilized in custom‐made conformal molds (Alpha Cradle; Smithers Medical Products, North Canton, OH) in the supine position with arms raised. A bellows system (Koninklijke Philips N.V., Amsterdam, Netherlands) was used to monitor patient breathing at simulation. No abdominal compression device was applied. Intravenous contrast was given before the DIBH CT scan; a second DIBH CT scan was usually obtained in a later contrast phase. Based on target visibility, one of the DIBH scans was selected for treatment planning. A free‐breathing scan was also performed for patient setup.

### 2.3 Treatment planning

The CT datasets were transferred to the treatment planning system Eclipse (Varian Medical Systems). GTVs were delineated in the patients’ DIBH CT data in MIM (MIM Software Inc., Cleveland, OH). The PTVs were created with 3–5 mm margins and edited as necessary to exclude normal tissue.^8^, ^9^ No internal target volume was required because the contours were created on DIBH scans.^26^ During the planning process, the fiducial markers were identified for later comparison. The planning objectives included normal tissue constraints (Table 2) and PTV coverage (low‐dose PTV *V*_100%_ ≥ 95%, high‐dose PTV *V*_100%_ ≥ 90% and *D*_max_ ≤ 110%). Our GI organ at risk (OAR) dose limits are biologically equivalent to reported conventional fractionation limits; bowel dose limits are based on reference.^27^ In most cases, all planning objectives were achieved with two or three arcs; however, more arcs were added when necessary.

**TABLE 2:** Normal tissue constraints^a^

		10 fx	15 fx	25 fx
Small bowel	*D*max	38 Gy	40 Gy	55 Gy
Stomach	*D*max	40 Gy	45 Gy	60 Gy
Duodenum	*D*max	40 Gy	45 Gy	60 Gy
Large bowel	*D*max	45 Gy	50 Gy	65 Gy
Liver (non‐GTV)	*DV*−700 cc	20 Gy	24 Gy	28 Gy
	*D*mean	20 Gy	24 Gy	28 Gy
Kidneys	*V*20 Gy	50%	50%	50%
Single functioning kidney	*V*20 Gy	33%	30%	30%

Abbreviation: GTV, gross tumor volume.

^a^ One or more of these constraints can be exceeded at the discretion of the attending physician.

### 2.4 Treatment delivery and intrafractional imaging

**FIGURE 1:**
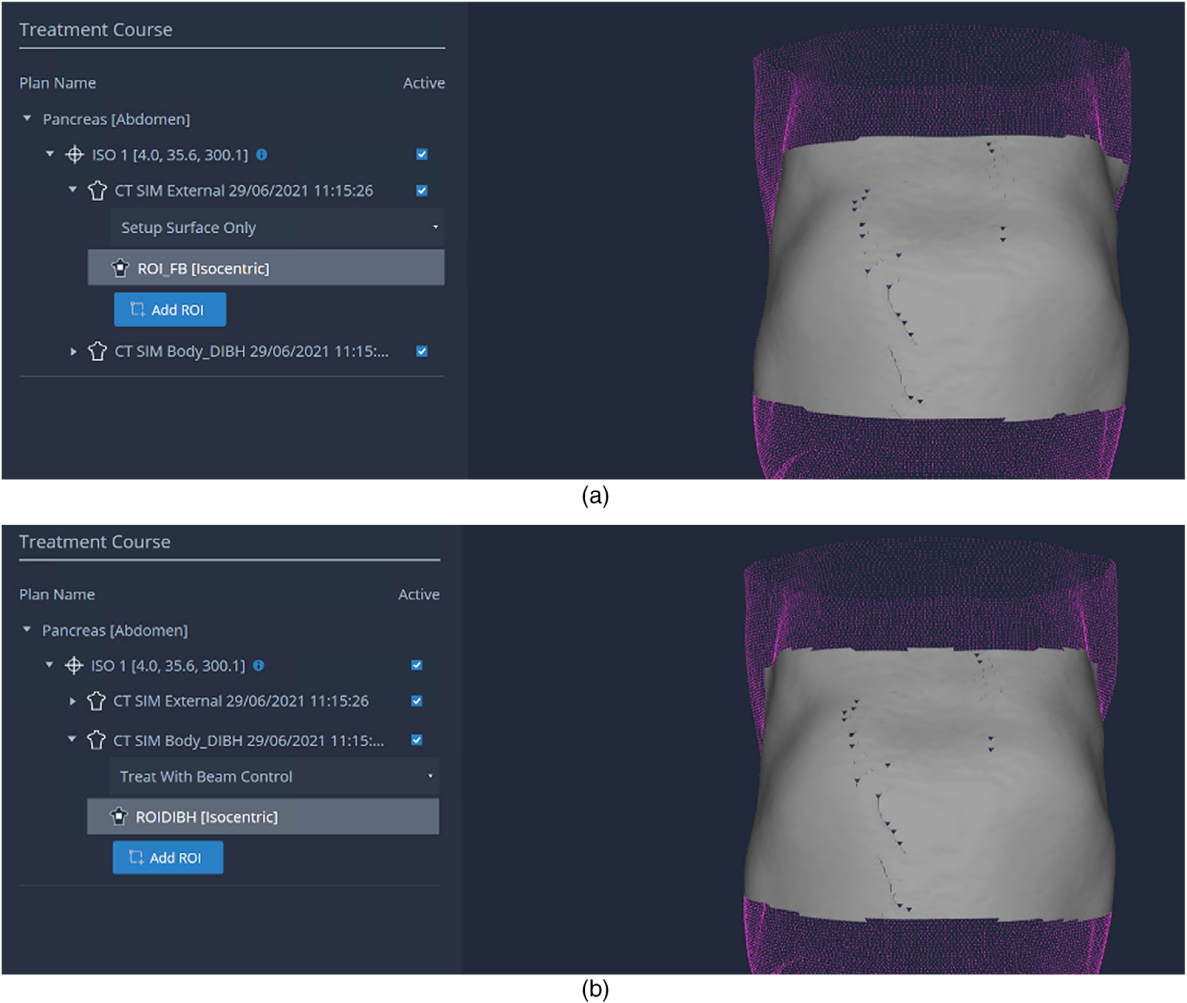
The region of interest for free breathing (a) and deep inspiration breath hold (b)

All treatments were performed on TrueBeam linacs (Varian Medical Systems) using the surface imaging system AlignRT (Vision RT, London, UK). The AlignRT system monitored a three‐dimensional (3D) region of interest (ROI) with a threshold of 3‐mm translation and 3° rotation. The ROI was at least 20 cm × 15 cm and covered the patient's abdomen (Figure 1). The free‐breathing scan was used for initial setup, while the DIBH scan was used for setup refinement and breathing motion monitoring. Cone beam CT (CBCT) scans were acquired, while the patient was instructed to perform DIBH to obtain the full volumetric datasets under breath‐hold. AlignRT does not gate the CBCT automatically; so, the kV beam was enabled and disabled manually when the patient's respiration entered and exited the respiratory window. Then, the CBCT images were aligned with the planning DIBH CT with marker location matched. The couch was moved accordingly, and a new reference surface was captured in AlignRT immediately after. During treatment, patients were instructed to perform voluntary DIBH in order to maintain the AlignRT ROI within the ± 3 mm and ± 3° gating window. If the patient's breathing was outside the gating window during treatment, the megavolt beam would be automatically turned off until the AlignRT ROI returned to the gating window.

Kilovolt image acquisition was triggered every 20° or 40° gantry rotation when MV beam was on. The time between real‐time kilovolt image acquisitions was approximately 10–15 s, compared to 5–6 s in other studies.^23^, ^24^ All kilovolt images were automatically saved to the image review system. Typically, about 27 images would be obtained for each fraction.^25^

### 2.5 Motion analysis

The previously described markers in the CBCT images were identified, and the 3D coordinates were recorded for comparison with the marker locations in the triggered images. A typical marker was approximately 5‐mm long, measured by CBCT, and the stent was about 5 cm. The number of distinct markers varied from patient to patient. If multiple markers were available, one of them was chosen according to the discernibility and relevance to the target. If a stent was designated as the image guidance alignment landmark, it would also be used in this study. The same marker was used for each patient throughout all image analyses.

Each triggered kilovolt image was carefully reviewed retrospectively in Offline Review Workspace (Varian Medical Systems), and the marker was identified in every frame. During VMAT delivery, the position of the marker in the kilovolt images changed as the gantry rotated. Pixel locations of both ends of the marker (or stent) in the planar images were recorded manually, and a spreadsheet (Microsoft Excel, Microsoft Corporation, Redmond, WA) was used to calculate the center location based on the average of the two ends.

With each one planar image, we can only determine the position in two directions, one being superior‐inferior (SI) direction and the other being the direction of the linear velocity of the imager during rotation. However, since the SI movement is usually the maximal in all three directions, according to reports for pancreatic tumor movement,^24^, ^28^, ^29^, ^30^, ^31^ we believe that SI coordinates alone were sufficient to characterize the pancreatic tumor motion. Taking into account the geometric magnification from the imaging projection, the pixel locations were converted to room coordinates according to the source and imager positions.^25^ For detailed description, see Supporting Information.

Thus, obtained was a time series of observed *internal* positions in the SI direction *z*_0_ *, z*_1_ *, z*_2_ and so forth at instants when the external signal (AlignRT) was within the gating window (± 3 mm– ± 3°), where *z*_0_ was the aligned position in the CBCT. We defined the target residual motion as *z*_max_−*z*_min_, while the *displacement vector* was defined as *z_i_*−*z*_0_ for *i* = 1, 2, and so forth

### 2.6 Treatment time

For treatment efficiency analysis, we obtained the treatment time per session from the imaging and treatment timestamps in the record and verify system (ARIA Oncology Information System, Varian Medical Systems). The treatment time for each session was calculated as the time since the first setup image to the conclusion of the last treatment field. This definition of treatment time includes the time it takes to image followed by adjustment of setup, interventions to reposition the target, and actual delivery of the treatment fields. Therefore, it is a measure of the efficiency of the treatment room usage. The scheduled duration was 30 min for each of those appointments.

## 3 RESULTS

**FIGURE 2:**
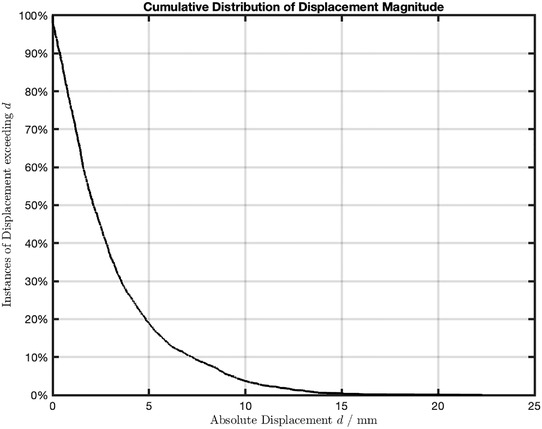
Cumulative distribution of displacement magnitude of all images analyzed

The motion characteristics of all fourteen patients, that is, residual motion and displacement, and session time are listed in Table 3. The residual motion reflects target position stability or precision of the target positioning. Displacement is the distance from the setup position during treatment, and it is related to the beam aiming accuracy. For the cohort, the average displacement of target relative to the initial position did not exceed 5 mm. Eighty‐one percent of the analyzed images have instantaneous displacement magnitudes within 5 mm, and 64% of them were within 3 mm (Figure 2). The mean magnitude of displacement over all patients was 1 mm with a standard deviation (SD) of 2 mm. Treatment time, including imaging time, was 17 min on average with an SD of 4 min.

**TABLE 3:** Intrafraction accuracy metrics and session time for all patients

Patient	Residual motion/mm	Displacement/mm	Fraction of images with	Session time/min
number	Average (range)	Average ± SD	displacement > 5 mm	Average ± SD
1	14 (8–19)	0 ± 1	17%	20 ± 6
2	14 (6–24)	0 ± 3	7%	15 ± 7
3	5 (4– 8)	0 ± 1	1%	11 ± 1
4	3 (3– 3)	−1 ± 2	0%	21 ± 15
5	10 (5–12)	0 ± 2	5%	11 ± 4
6	21 (13–29)	3 ± 2	54%	20 ± 6
7	4 (4– 4)	−3 ± 1	1%	9 ± 1
8	7 (6–11)	0 ± 3	12%	17 ± 11
9	12 (9–13)	0 ± 5	28%	22 ± 7
10	11 (7–13)	−2 ± 1	6%	18 ± 7
11	5 (4– 7)	1 ± 3	12%	23 ± 11
12a^a^	8 (6–10)	2 ± 4	26%	13 ± 5
12b^a^	11 (11–12)	3 ± 2	32%	15 ± 3
13	14 (10–16)	5 ± 4	55%	17 ± 6
14	9 (8–10)	1 ± 3	9%	21 ± 4
Average	10 (3–21)	1 ± 2	19%	17 ± 4

*Note*: Positive displacement indicates the superior direction.

Abbreviation: SD, standard deviation.

^a^ Patient 12 was treated separately for two liver metastases.

**FIGURE 3:**
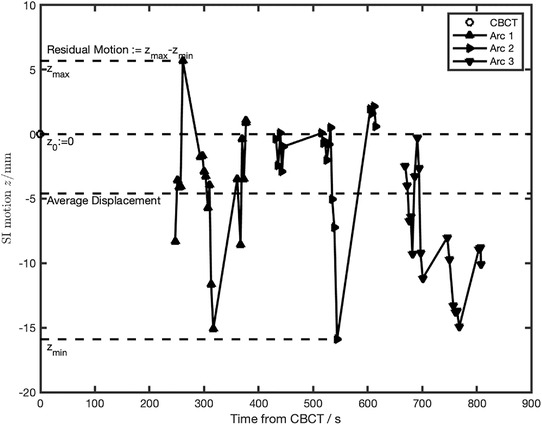
The superior‐inferior (SI) movement of the marker during a treatment session of one patient (Patient 6), starting at the time of cone beam computed tomography (CBCT). Significant residual motion was present in that session

Although the external surface was limited to ± 3 mm translation and ± 3° rotation, significant instantaneous residual motion of the internal target was observed in some patients, ranging from 3 mm to about 2 cm. The cohort‐averaged residual motion was 10 mm, with SD of 5 mm. As an example of significant residual motion, Figure 3 displays the change in position for Patient 6 during one session of DIBH treatment.

Patient 6 exhibited the largest residual motion. The largest average displacement of 5 mm was observed in Patient 13, who also exhibited the largest portion of instances with > 5‐mm displacement, compared to the 5‐mm PTV expansion. Those values implied poor correlation between external surface and internal motion for those patients. The fraction of images showing displacement > 5 mm correlated highly with both the residual motion and the average displacement (*p* = 10^–6^ and 10^–3^, respectively; paired *t*‐test).

## 4 DISCUSSION

For highly conformal, ablative radiation treatment, which has rapidly been adopted for treating primary and metastatic tumors, the ability to accurately localize the correct target and deliver treatment safely is at least as important as the technical planning capabilities.^10^ Recent technological advancements and innovative solutions for managing internal organ motion have enabled safe radiotherapy dose escalation.^11^ In the present study, we assessed internal target movement during DIBH radiation therapy of GI cancer with surface image guidance. Although the surface image guidance restricted the external ROI motion to ± 3 mm– ± 3°, it was often found that the internal movement amplitude was greater, about 10 mm in average, and in extreme cases 20 mm. This is consistent with other studies,^14^, ^15^, ^16^, ^32^, ^33^ which underlines the necessity of real‐time monitoring of the internal target.

Our current study acquired, and retrospectively analyzed, kilovolt images of internal targets during treatment beam delivery; however, internal target position was *not* quantified in real‐time during treatment. Therapists were instructed to monitor the triggered images and pause treatment if large displacements were indicated in two consecutive images. However, that was a manual process and involved some subjectivity. Furthermore, the “Auto Window/Level” of the system may not be optimal in visualizing the relevant marker.

Since each triggered image indicated the target position at one instance during one particular treatment session, part of the target may not be encompassed by the PTV at those instances when displacement was greater than the PTV margin. Despite 19% of images showed > 5‐mm displacement, the average displacement was approaching zero for several patients (Table 3). However, nonzero average displacement (up to 5 mm) was indicated for the other patients studied, implying drift of the displacement during treatment. For those patients, AlignRT *did not* indicate the true internal motion, and thus large displacement would have gone unnoticed if radiographic means of monitoring was not present. For the whole cohort, the average displacement was only 1 mm, which indicated that the mean target position during treatment was very close to the initial position established during CBCT setup.

Dawson et al. investigated the reproducibility of organ movement in eight patients with liver cancer and suggested the need for daily online imaging.^34^ Recently, we used kilovolt images triggered during each DIBH treatment session to track internal targets throughout the treatment course, where the DIBH was facilitated by the RPM system.^25^ As discussed in reference,^25^ for a typical patient, high‐dose PTV *D*_95%_ decreased by approximately 5% with an SI offset of 3 mm; > 10% with a 5‐mm offset; and > 20% with a 10‐mm offset. For Patient 6 in this study, 3‐mm superior displacement leads the high‐dose PTV *D*_95%_ to drop from 99% to 97%; for Patient 13, 5 mm superior displacement would cause the high‐dose PTV *D*_95%_ to drop from 97% to 91%. Ding et al. determined limits on motion of pancreas for SBRT.^35^ A conservative limit of 6 mm was deemed safe for all 91 patients in their study if they were treated to 33 Gy in five treatments. If the dose was increased to 50 Gy, the limit would be reduced to 4.2 mm.

We compared the current cohort with our previous DIBH cohort of the same disease site but using Varian RPM system instead of surface image guidance.^25^ The residual motion of the SGRT cohort turned out slightly greater than that of the RPM cohort (*p* = 0.07; Mann‐Whitney *U* test). For the RPM cohort, > 5‐mm displacement was observed in ∼10% of the images analyzed, compared to 19% in the current SGRT cohort. Four patients (27%) in this cohort exhibited at least 3‐mm displacement, compared to 12% in the RPM cohort, and one case was 5 mm (7%), compared to none in the RPM cohort.^25^ Note that the same triggered kilovolt imaging scheme was used for both cohorts. All other reported characteristics (age, gender, displacement, and session time) were statistically comparable (*p* > 0.1). For our RPM cohort, the external gating window was set to 3 mm in one dimension (± 1.5 mm).^25^ For the current SGRT cohort, ± 3 mm ‐ ± 3° corresponds to a window of 6 mm–6° for all six degrees of freedom, which may be less restrictive and may have contributed to the greater residual motion. If we attempted using tighter gating windows for AlignRT, the treatment sessions may likely take longer (than using the RPM technique), and patient comfort may be impacted. The reported session time using RPM was 15 min (SD 3 min).^13^ Furthermore, the accuracy of the AlignRT system may also have been affected by the extended kilovolt source and imager during treatment. Triggered kilovolt imaging requires both the kilovolt source and imager being extended throughout the treatment. During the gantry rotation of arc treatments, the treatment gantry head, kilovolt source, and kilovolt imager may block some camera angles of the AlignRT system.

While AlignRT does not issue a warning when one of the three cameras was blocked, it would hold the treatment beam when two cameras were blocked. If that happened to a particular patient, we were not able to use triggered kilovolt imaging for that patient. As a result, simultaneous application of AlignRT and triggered kilovolt imaging was not guaranteed for all cases. In this study, we could only include patients for whom AlignRT and triggered kV imaging were successfully applied simultaneously. And all kilovolt images reported in this study were acquired when AlignRT determined that the ROI was within tolerance relative to the reference DIBH surface.

One limitation of the current study is the somewhat arbitrarily selected rectangular ROI for AlignRT (Figure 1), which may not be optimal. On the other hand, the RPM system monitors only a very specific region with dimensions of 3–5 cm. While the RPM block was also placed on the patient's abdomen in our previous study,^25^ we are currently investigating using more specific ROI excluding the parts of surface which are not affected by breathing motion. The preliminary result will be published in a subsequent report. Another limitation is the lack of real‐time position information of the OARs. With real‐time monitoring of targets over different angles during VMAT treatment, if we can ensure reasonable target localization accuracy on‐treatment, it is unlikely that the surrounding organs would enter the treatment field significantly and systematically.

Our kilovolt imaging data only allowed consistent determination of marker positions in the SI direction. Any finite motion in another direction would only increase the residual motion. Therefore, the reported values are an underestimation, even though the SI component accounted for most of the motion. Based on the large residual motion observed in this study, as well as several known limitations of the surface imaging system, the practice of SGRT for GI targets should be exercised with extra care. Radiographic monitoring of *internal* targets during treatment is essential to determine the intrafraction motion. In order to ensure safe treatment, therapist intervention with repeated setup imaging and couch correction may be necessary if target moves beyond tolerated range. In the current study, the system of triggered imaging did not display quantitative information during treatment. We have developed a system to report displacement values in real‐time for prostate fiducial markers.^36^ We are actively working on expanding that system to other anatomical sites such as lung, spine, and abdomen.^37^

## 5 CONCLUSION

In conclusion, we found that the internal target movement can be significantly different from the external surrogate movement in DIBH radiation therapy, even when we were using surface image guidance. Precaution is needed when applying surface image guidance for GI cancer treatment. Using surface image guidance as a solo technique for real‐time motion monitoring (during delivery of treatment beams) is discouraged when the correlation between internal anatomy and patient surface is limited. Real‐time radiographic verification of the target location is critical to ensure safe delivery of radiation therapy.

## CONFLICT OF INTEREST

The authors have nothing to disclose.

## AUTHOR CONTRIBUTIONS

*Concept and design*: Chuan Zeng, Wei Lu, Xiang Li, Tianfang Li. *Acquisition, analysis, or interpretation of data*: Chuan Zeng, Wei Lu, Marsha Reyngold, John J. Cuaron, Xiang Li, Laura Cerviño, Tianfang Li. *Drafting of the manuscript*: Chuan Zeng, Tianfang Li. *Critical revision of the manuscript for important intellectual content*: Chuan Zeng, Wei Lu, Laura Cerviño, Tianfang Li. *Statistical analysis*: Chuan Zeng. Obtaining funding: Wei Lu, Xiang Li, Tianfang Li. *Administrative, technical, or material support*: Chuan Zeng, Wei Lu, Laura Cerviño, Tianfang Li. *Supervisions*: Chuan Zeng, Wei Lu, Xiang Li, Laura Cerviño, Tianfang Li. Chuan Zeng had full access to all the data in the study and takes responsibility of the integrity of the data and the accuracy of the data analysis.

## Supporting information



Supporting Information
