# Supplementary material for: Intrafractional accuracy and efficiency of a surface imaging system for deep inspiration breath hold during ablative gastrointestinal cancer treatment
Source: J Appl Clin Med Phys. 2022 Jul 30;23(11):e13740. doi: 10.1002/acm2.13740 (PMC9680575; doi:10.1002/acm2.13740)
Supplement: Supplementary file 1 — Supporting Information [file ACM2-23-e13740-s001.pdf]

Intrafractional accuracy and efficiency of a surface imaging system for deep inspiration breath hold during ablative gastrointestinal cancer treatment

(Dated: July 18, 2022)

SUPPLEMENTAL MATERIAL

Geometric magnification from the X-ray projection

As shown in Fig. S1, the superior-inferior (SI) motion Δz for point $P(x, y, z)$ in patient is magnified in the imaging plane by a factor of $\frac{\overline{SI}}{\overline{SP_0}}$, where $\overline{SI}$ is commonly denoted as source-to-imager distance (SID). Simple geometry leads to

$$\overline{SP_0} = \overline{SO} - x \sin\alpha + y \cos\alpha, \tag{1}$$

in which $\overline{SO}$ is commonly referred to as source-to-axis distance (SAD), and α is the angular position of the kV source (gantry angle). Thus, the actual SI motion Δz is obtained from the SI motion in image $\Delta z'$ as

$$\Delta z = \Delta z' \frac{\mathrm{SAD} - x \sin\alpha + y \cos\alpha}{\mathrm{SID}}. \tag{2}$$

In our treatment rooms, SAD is 100 cm and SID is 150 cm.

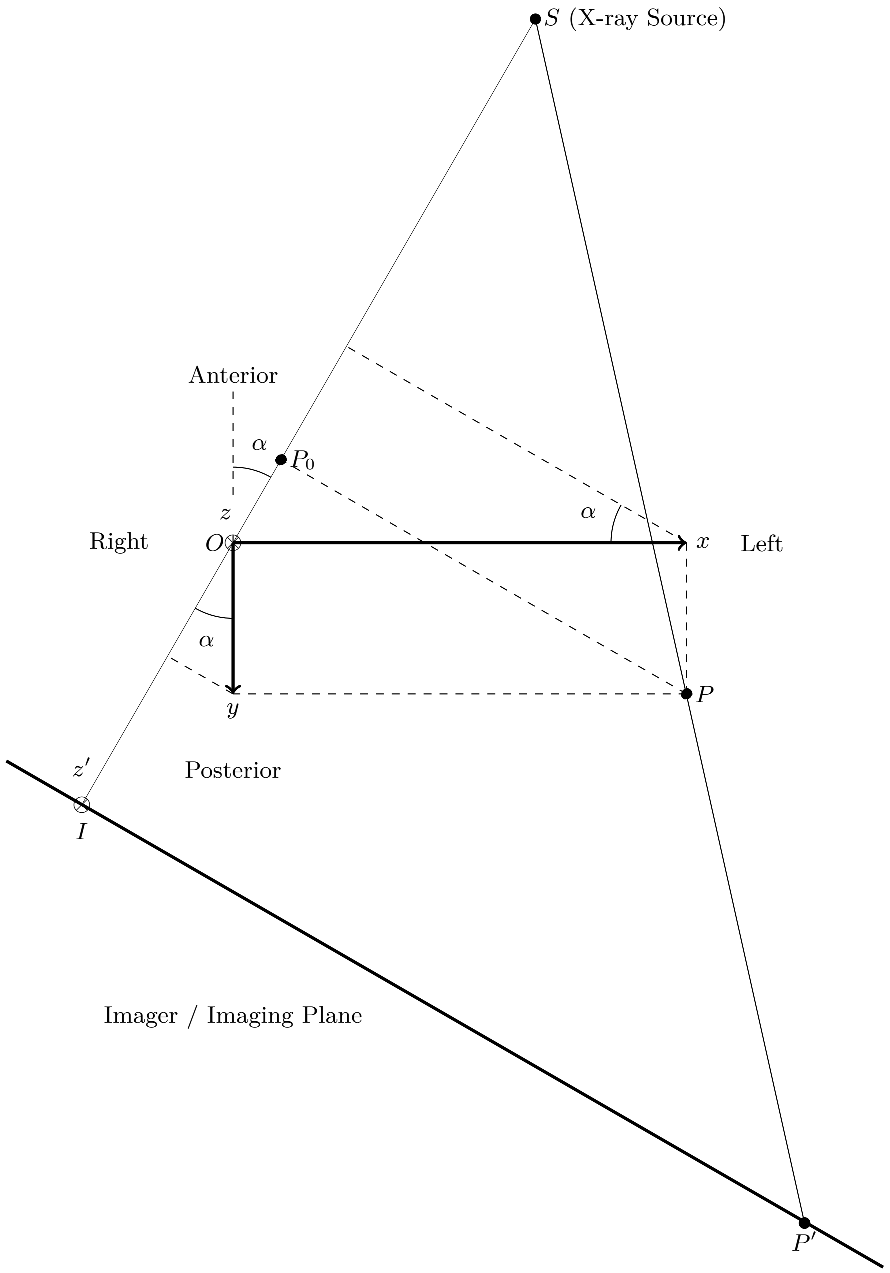

FIG. S1: Translate the pixel information of the marker on a kV image (P') to room coordinates (P) by backprojecting the marker to the plane where it was initially localized during setup cone beam computed tomography. In this axial view, kV source S is at angle α. The central axis of the kV beam passes through isocenter O and intersects with the imager plane at point I. The offsets x and y from the isocenter have been exaggerated for the purposes of illustration. The z-axis points into the page.
